# Comparison of the inhibitory effects of *Lactobacillus* supernatant and coculture on *Gardnerella vaginalis*

**DOI:** 10.1186/s13104-025-07414-w

**Published:** 2025-08-09

**Authors:** Ferenc Somogyvári, Nándor Tűzkő, Attila Kereszturi, László Párducz, Mária Szécsényi, Valéria Endrész, Marianna Ábrók, Caleb M. Ardizzone, Katalin Burián, Dezső Péter Virok

**Affiliations:** 1 Department of Medical Microbiology, Albert Szent-Györgyi Health Center and Albert Szent-Györgyi Medical School, University of Szeged, Semmelweis str. 6, Szeged, H-6725 Hungary; 2 Department of Obstetrics and Gynecology, St. Margaret’s Hospital, Budapest, H-1032 Hungary; 3 Department of Obstetrics and Gynecology, Albert Szent-Györgyi Health Center, Faculty of Medicine, University of Szeged, Semmelweis str. 1, Szeged, H-6701 Hungary; 4 Department of Obstetrics and Gynecology, Pándy Kálmán County Hospital, Semmelweis str. 1, Gyula, H-5700 Hungary; 5 Division of Infectious Diseases, Department of Medicine, Indiana University School of Medicine, 635 Barnhill Drive, MS 258, Indianapolis, IN 46202 USA

**Keywords:** *Lactobacillus*, *Gardnerella*, Vaginosis, DNA, Direct, qPCR, Growth, Antimicrobial, Supernatant

## Abstract

**Objective:**

Vaginal lactobacilli play a crucial role in inhibiting bacteria such as *Gardnerella vaginalis* (*G. vaginalis*), a key contributor to dysbiosis and bacterial vaginosis. We aimed to compare the inhibitory effects of *Lactobacillus* cell-free supernatants on *G. vaginalis* growth with those observed in *Lactobacillus*-*G. vaginalis* coculture, an experimental setup that more closely mimics in vivo conditions.

**Results:**

To identify the optimal medium for coculture experiments, MRS broth and NYC-III broth were compared. NYC-III significantly enhanced the growth of *G. vaginalis* and four of the five tested *Lactobacillus* strains. We then developed a direct quantitative PCR (qPCR) method that allowed us to specifically measure *G. vaginalis* genome concentrations in cocultures with *Lactobacillus*. This direct qPCR did not require DNA extraction and had a 4,096-fold dynamic range. We then assessed the inhibition of *G. vaginalis* growth by *Lactobacillus* cell-free supernatants in *G. vaginalis* cultures by measuring optical density and the *Lactobacillus*-mediated inhibition in cocultures by the *G. vaginalis* specific direct qPCR. The two measurement methods showed different levels of inhibitory activity for two of the five *Lactobacillus* strains tested. These findings suggest that coculture experiments should be conducted in place of, or in addition to, supernatant-based inhibitory assays.

## Introduction

*Lactobacillus* species play a crucial role in maintaining a healthy vaginal microbiota by producing lactic acid, hydrogen peroxide, and bacteriocins [1], which inhibit the overgrowth of pathogenic bacteria. A decrease in *Lactobacillus* abundance is a hallmark of bacterial vaginosis (BV), where the protective lactobacilli are outnumbered by other bacteria in the vaginal microbiota. The socioeconomic impact of bacterial vaginosis is significant, with a prevalence ranging from 23 to 29% in the general population [2]. *G. vaginalis* is a Gram-variable bacterium recognized as a key player in the pathogenesis of BV, often found in high abundance in affected women [3, 4]. *Lactobacillus* can effectively inhibit *G. vaginalis*, and the presence of *Lactobacillus* species is often inversely correlated with the abundance of *G. vaginalis* in vaginal samples [5].

Screening the antimicrobial activity of vaginal probiotic candidates against relevant microorganisms, including *G. vaginalis*, is a crucial step in the selection process. A common method is testing the inhibition of *Lactobacillus* cell-free supernatants on relevant pathogens, such as *G. vaginalis* [6]. While this method is relatively straightforward, it fails to replicate the natural interactions between lactobacilli and *G. vaginalis* that occur in vivo within a shared microenvironment. Coculture methods, in which *Lactobacillus* strains are incubated with *G. vaginalis*, can address this issue. A challenge with these approaches is the selective measurement of *G. vaginalis* growth against the *Lactobacillus* background. For example, Pessoa et al. cultured *Lactobacillus* isolates with *G. vaginalis* in BHI broth, made a dilution series, and plated them on solid agars to count the *G. vaginalis* colonies [6]. The identification and manual counting of *G. vaginalis* colonies were based on colony morphology. Although this is a well-established method [6, 7], it requires considerable manual labor, making it less suitable for screening. The alternative is the specific quantitation of *G. vaginalis* genome by qPCR in the coculture samples [8]. However, the cost and labor of the DNA extraction can be significant, making this approach also impractical for screening.

Previously, we developed a straightforward, DNA extraction-free direct qPCR method to specifically measure the growth of *Chlamydia trachomatis* [9] and herpes simplex virus-2 [10] in vitro. In these studies, direct qPCR was effective for measuring the antimicrobial activity of various established and novel compounds. Here, we applied the direct qPCR method to measure *G. vaginalis* growth in coculture with *Lactobacillu*s and found that *Lactobacillus*-mediated inhibition differed markedly from that observed with *Lactobacillus* cell-free supernatants.

## Materials and methods

### Bacterial strains

Two *Lactobacillus crispatus* (*L. crispatus*-200, *L. crispatus*-202), two *L. gasseri* (*L. gasseri*-212, *L. gasseri*-224), one *L. jensenii (L. jensenii*-241), and one *G. vaginalis* isolate were obtained during a routine microbiology diagnosis of vaginal swab samples (Department of Medical Microbiology, University of Szeged, Szeged, Hungary) and were used in this study. Species-level identification was performed using the MALDI Biotyper^®^ sirius/ MALDI Biotyper^®^ microflex LT (Bruker, Bremen, Germany).

### Growth kinetics of *G. vaginalis* and *Lactobacillus* strains

*Lactobacillus* isolates and *G. vaginalis* were cultured (*n* = 4) in 200 µl of either Man–Rogosa–Sharpe (MRS) medium (Bio-Rad, Hercules, CA, USA) or NYC-III medium (ATCC Medium 1685 [11]) for 72 h at 37 °C with 5% CO_2_. The initial densities were adjusted to 0.1 OD_600_. OD_600_ was measured at 0, 2, 4, 6, 8, 24, 48, and 72 h for *G. vaginalis*, and at 0, 24, 48, and 72 h for *Lactobacillus* spp., using an EZ Read 400 Microplate Reader (Harvard Bioscience, Holliston, MA, USA).

### Direct qPCR of *G. vaginalis*

Direct qPCR of *G. vaginalis* was conducted using HOT FIREPol EvaGreen qPCR Supermix (Solis Biodyne, Tartu, Estonia) in a Bio-Rad CFX96 real-time PCR System (Bio-Rad, Hercules, CA, USA). The initial qPCR experiments utilized four primer pairs previously documented in the literature [12–15] and were named Primer-1, Primer-2, Primer-3 and Primer-4. Following a preliminary sensitivity screen, the primer pairs (Primer-3) from Zozaya-Hinchliffe et al. were selected [14], with the primer sequences: 5’-GGAAACGGGTGGTAATGCTGG-3’, 5’-CGAAGCCTAGGTGGGCCATT-3’. The qPCR mixture contained 2 µl HOT FIREPol EvaGreen qPCR Supermix, 1 µl forward and reverse primers (10 pmol each), 1 µl template, and 5 µl Milli-Q water, totaling 10 µl. The qPCR started with a 12 min activation step at 95 °C, followed by 40 cycles consisting of 95 °C for 15 s, 68 °C for 25 s, and 72 °C for 20 s, after which fluorescence was measured. Genomic DNA from *G. vaginalis* was extracted using the Zymo Research Quick-DNA Miniprep kit (Zymo Research, Irvine, CA, USA) and used as a comparison to the direct qPCR.

### Inhibition of *G. vaginalis* growth by *Lactobacillus* supernatant

*Lactobacillus* cultures (10 µl), adjusted to 0.1 OD_600_, were inoculated into 1 ml of NYC-III medium and incubated for 48 h at 37 °C with 5% CO_2_. After incubation, the supernatants were obtained by harvesting the cells through centrifugation for 15 min at 8,000 × g and filtered using 0.22 μm Millex-GS Filter Units (St. Louis, MO, USA). *G. vaginalis* (0.1 OD_600_) was cultured in NYC-III medium with 50%, 25%, and 12.5% v/v *Lactobacillus* cell-free supernatants for 48 h at 37 °C with 5% CO_2_. OD_600_ was measured at 48 h (*n* = 3). Statistical comparisons were performed using a one-way ANOVA with a significance threshold of *p* < 0.05. Complete hierarchical clustering of inhibition data was performed by SRplot [16].

### Inhibition of *G. vaginalis* growth by *Lactobacillus* coculture

*Lactobacillus* isolates and *G. vaginalis* were propagated separately in NYC-III medium for 24 h at 37 °C with 5% CO_2_. Initial OD_600_ values were adjusted to 0.1 or 0.01. *Lactobacillus* isolates were mixed with *G. vaginalis* at ratios of 10:1, 1:1, and 1:10. *Lactobacillus* and *G. vaginalis* cocultures were incubated for 48 h at 37 °C with 5% CO_2_. Direct qPCR was utilized to quantify the *G. vaginalis* concentrations in the culture medium (*n* = 4). Statistical comparisons of qPCR cycle threshold (Ct) values between coculture and monoculture *G. vaginalis* samples were performed using a Student’s t-test, with a significance threshold of *p* < 0.05 as previously described [17]. Complete hierarchical clustering of inhibition data was performed by SRplot [16].

## Results

**Fig. 1:**
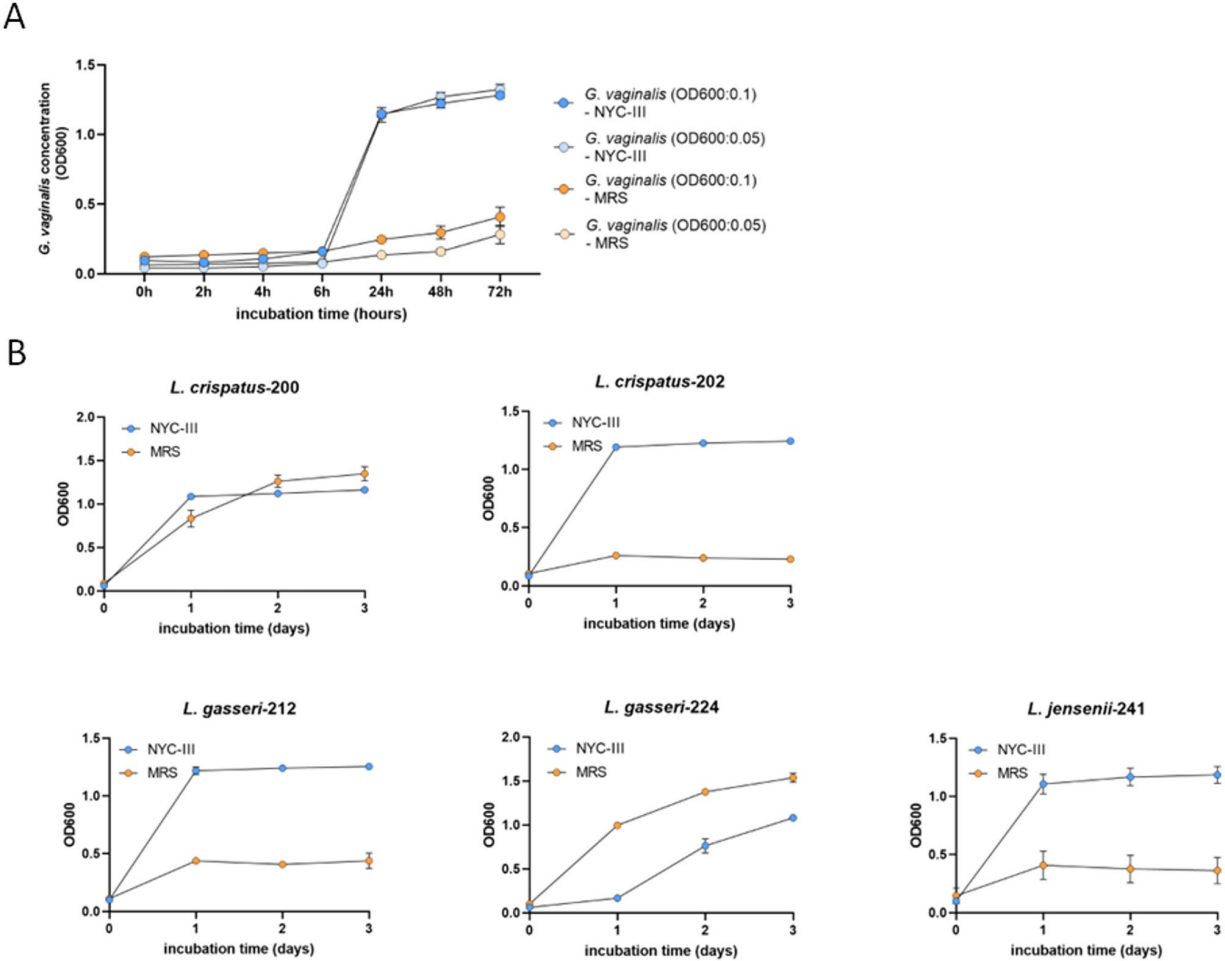
Growth kinetics of *G. vaginalis* and *Lactobacillus* spp. in MRS and NYC-III media. (**A**) *G. vaginalis* growth in MRS and NYC-III media. *G. vaginalis* was propagated in MRS or NYC-III media for 72 h at 37 °C with 5% CO_2_. (**B**) *Lactobacillus* spp. growth in MRS and NYC-III media. *Lactobacillus* strains were propagated in MRS or NYC-III media for 72 h at 37 °C with 5% CO_2_. OD_600_ was measured at each timepoint (*n* = 4). Data are presented as mean ± SD

### Growth kinetics of *G. vaginalis* and *Lactobacillus* spp. In MRS and NYC-III media

To compare the ability of MRS and NYC-III media to support *G. vaginalis* and *Lactobacillus* growth, we cultured both bacteria in parallel in each medium (Fig. 1A). *G. vaginalis* growth was followed using two initial concentrations, 0.1 OD_600_ and 0.05 OD_600_. *G. vaginalis* growth was limited after 6 h culture in both media, indicating that the bacterium was in the lag phase. However, there was a dramatic difference at the 24 h timepoint. *G. vaginalis* reached ~ 1.14 OD_600_ in NYC-III, while only minimal growth could be detected in MRS. After 72 h incubation in NYC-III medium, *G. vaginalis* concentrations increased to approximately 1.3 OD_600_, regardless of the initial inoculum size, whereas MRS only supported minimal growth of *G. vaginalis*, reaching 0.283 to 0.408 OD_600_, depending on the initial inoculum size. These data indicate that MRS may not support the growth of a low amount of *G. vaginalis*. *Lactobacillus* growth was also tested in the two media (Fig. 1B). Interestingly, only *L. gasseri*-224 grew better in the well-established *Lactobacillus* medium MRS than in NYC-III. *L. crispatus*-200 exhibited similar growth kinetics in both media, and the remaining three isolates grew better in NYC-III than in MRS. Altogether, these experiments showed that NYC-III supports the growth of both *G. vaginalis* and the *Lactobacillus* strains, therefore it was used for coculture experiments.

**Fig. 2:**
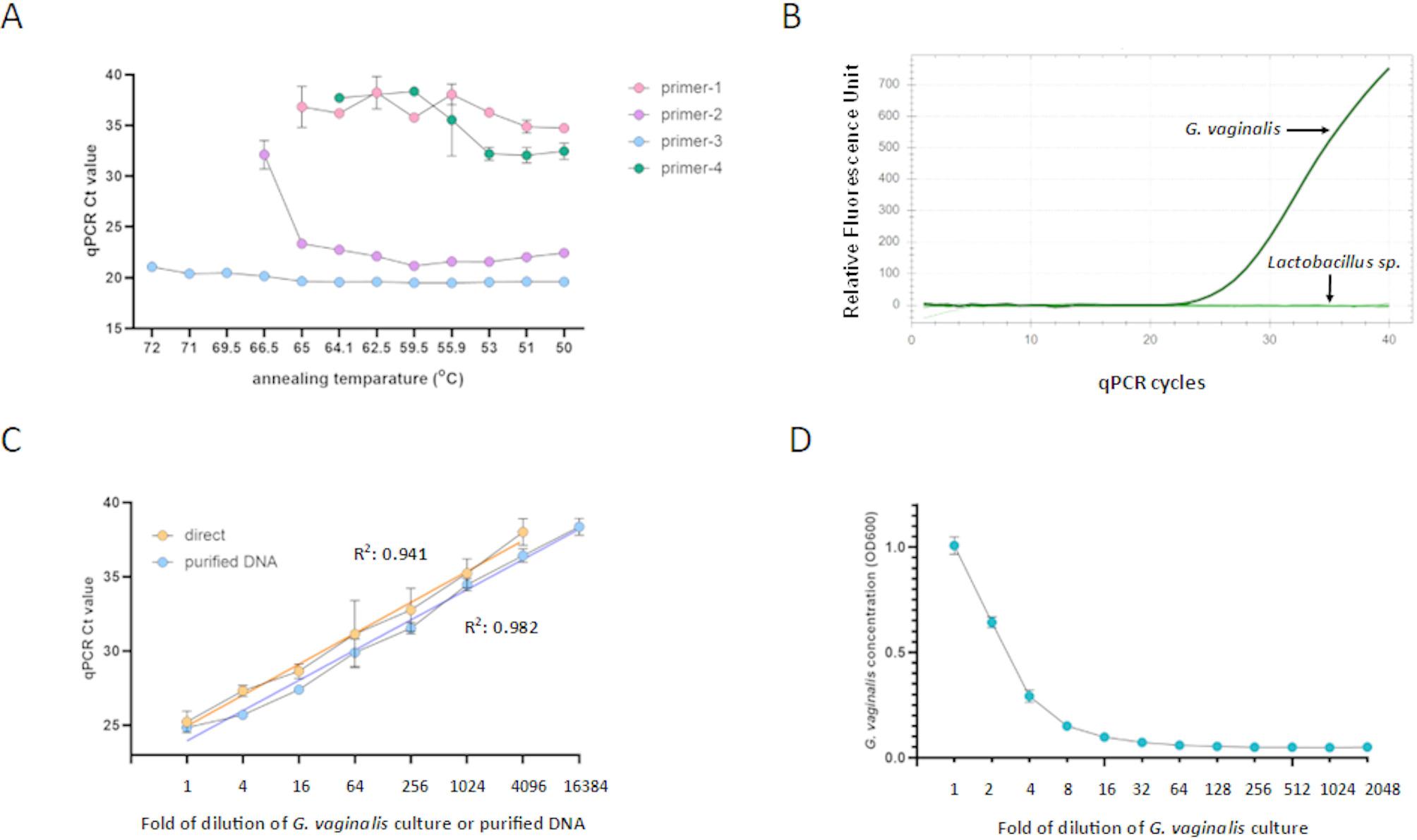
Development of a direct qPCR method to detect *G. vaginalis* in cocultures. (**A**) Impact of annealing temperature on the Ct values of *G. vaginalis* direct qPCR (*n* = 3). (**B**) Specificity of *G. vaginalis* direct qPCR. Cultures of *G. vaginalis* and individual *Lactobacillus* strains were used as templates in a *G. vaginalis*-specific direct qPCR. Representative amplification is shown. (**C**) Dynamic range of *G. vaginalis*-specific direct qPCR. Serial four-fold dilutions of *G. vaginalis* cultures and purified *G. vaginalis* DNA were used as templates in a qPCR (*n* = 3). Linear regression was calculated for both templates, with R² values shown. (**D**) Dynamic range of spectrophotometry of *G. vaginalis* concentration. *G. vaginalis* was propagated in NYC-III medium, and the concentration was adjusted to 1.0 OD_600_. Serial two-fold dilutions of the culture were performed, and the OD_600_ was measured (*n* = 3). Data are presented as mean ± SD

### Development of a *G. vaginalis*-specific direct qPCR

Four previously published *G. vaginalis*-specific primer pairs [12–15] were evaluated for their performance in a direct qPCR. NYC-III medium containing *G. vaginalis* (0.1 OD_600_) served as the template in the direct qPCR. HOT FIREPol EvaGreen qPCR Supermix was utilized in the qPCR, as this master mix had performed well in a direct qPCR assay previously [18]. The tested primer pairs exhibited significant differences in the sensitivity of *G. vaginalis* detection (Fig. 2A). Primer-1 and Primer-4 produced the highest Ct values, but neither of these primer pairs could detect the bacterium above a 65 °C annealing temperature. Primer-2 demonstrated considerably lower Ct values compared to Primer-1 and Primer-4. qPCR with primer-3 resulted in the lowest Ct values, and, interestingly, the Ct values were close to constant at higher annealing temperatures. Overall, Primer-3 produced the lowest Ct values, even at high annealing temperatures, and was selected for further qPCR using a 68 °C annealing temperature. Specificity of Primer-3 was demonstrated using NYC-III medium containing *G. vaginalis* or one of the five *Lactobacillus* strains (Fig. 2B). Primer-3 also exhibited a 4,096-fold dynamic range, using serial four-fold dilutions of *G. vaginalis* culture as direct template (Fig. 2C). When Ct values were plotted against the dilution factor, the linear regression slope was 2.083, indicating an average 4.3-fold concentration difference between the four-fold template dilutions. qPCR with purified DNA from *G. vaginalis* cultures demonstrated slightly higher sensitivity and a four-fold greater dynamic range than the direct qPCR. These data indicate that the performance of the direct qPCR was comparable to that of the regular qPCR with purified DNA template and omitting the DNA purification step is feasible and may significantly simplify growth measurements. Additionally, a serial two-fold dilution series of *G. vaginalis* culture revealed that spectrophotometry had a dramatically lower dynamic range, showing an approximately 4-fold dynamic range (Fig. 2D). While OD_600_ measurement cannot be used to measure *G. vaginalis* growth in a coculture, these data also demonstrate the poor dynamic range of spectrophotometry in comparison to qPCR.

**Fig. 3:**
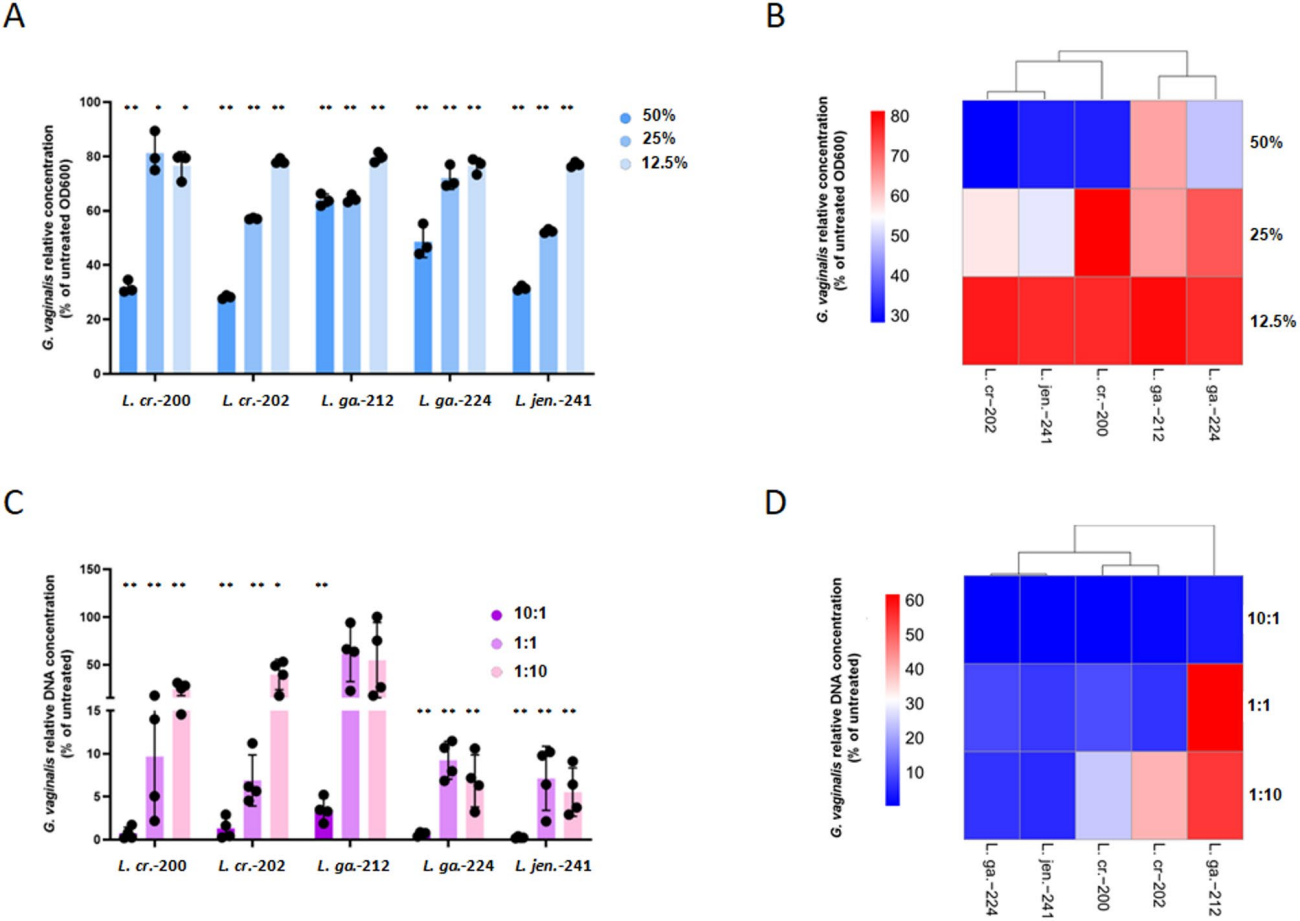
Comparison of methods for testing *Lactobacillus*-mediated inhibition of *G. vaginalis*. (**A**) Inhibition of *G. vaginalis* growth by *Lactobacillus* supernatants. *G. vaginalis* growth in NYC-III medium with 50%, 25%, and 12.5% v/v *Lactobacillus* supernatants for 48 h at 37 °C with 5% CO_2_. OD_600_ data are presented as mean ± SD. Statistical comparisons of OD_600_ values (*n* = 3) were performed using one-way ANOVA (*: *p* < 0.05, **: *p* < 0.01). (**B**) Comparison of the inhibitory effect of *Lactobacillus* supernatants was conducted through hierarchical clustering of the average *G. vaginalis* growth in the presence of *Lactobacillus* supernatants. (**C**) Inhibition of *G. vaginalis* growth in coculture with *Lactobacillus* spp. *G. vaginalis* and *Lactobacillus* strains were mixed in 10:1, 1:1, and 1:10 *Lactobacillus* sp./*G. vaginalis* ratios. Bacteria were cultured in NYC-III medium for 48 h at 37 °C with 5% CO_2,_ and a direct qPCR was applied to detect *G. vaginalis* growth (*n* = 4). Data are presented as mean ± SD. Statistical comparisons of Ct values between coculture and monoculture *G. vaginalis* were performed using a Student’s t-test (*: *p* < 0.05, **: *p* < 0.01). (**D**) Comparison of the inhibitory effect of *Lactobacillus* strains was conducted through hierarchical clustering of the average *G. vaginalis* growth in the coculture samples

### Comparison of methods for testing *Lactobacillus*-mediated inhibition of *G. vaginalis*

First, we employed the well-established cell-free supernatant-mediated inhibitory assay to evaluate the inhibitory phenotypes of the *Lactobacillus* strains. Cell-free supernatants from each strain were tested at 50%, 25%, and 12.5% v/v (Fig. 3A). At 50%, three strains reduced *G. vaginalis* growth to approximately 28.2–31.9% of the control, while *L. gasseri*-212 and *L. gasseri*-224 were less effective, only reducing growth to 48.6–63.9%. At 25%, *L. jensenii*-241 and *L. crispatus*-202 exhibited the greatest inhibition, limiting *G. vaginalis* growth to 52.5% and 57%, respectively. At 12.5%, all strains restricted growth to approximately 80% of the control. *Lactobacillus* strains were then clustered based on the inhibitory activity of their supernatants. *L. crispatus*-202 and *L. jensenii*-241 formed a high-inhibitor group, *L. gasseri*-212 and *L. gasseri*-224 formed a low-inhibitor group, and *L. crispatus*-200 fell in between (Fig. 3B).

For coculture experiments, we tested three initial *Lactobacillus*/*G. vaginalis* ratios: 10: 1, 1:1, and 1:10. The direct qPCR method was employed to monitor *G. vaginalis* growth. *L. jensenii*-241 and *L. gasseri*-224 significantly inhibited *G. vaginalis* growth at all three inoculum ratios (Fig. 3C). *L. crispatus*-200 and *L. crispatus*-202 showed marked growth inhibition only when there were either 10-fold more *Lactobacillus* or equal amounts of *Lactobacillus* in the coculture samples initially. *L. gasseri*-212 was the weakest inhibitor, exhibiting significant inhibition only when there was a 10-fold more *Lactobacillus* in the cocultures initially. *Lactobacillus* strains were then clustered based on their inhibitory activity in cocultures. *L. jensenii*-241 and *L. gasseri*-224 formed a high-inhibitor group, *L. crispatus*-200 and *L. crispatus*-202 formed a moderate-inhibitor group, and the weak-inhibitor *L. gasseri*-212 fell below (Fig. 3D).

The difference between coculture-based and supernatant-based inhibition tests was especially significant for *L. crispatus*-224, which showed poor inhibition in the supernatant-based assay but proved to be an effective inhibitor in the coculture assay. The opposite was true for *L. crispatus*-202, which showed good inhibition in the supernatant-based assay but was a less effective inhibitor in the coculture assay. These differences are not unexpected. In vitro, both measurement methods primarily assess secreted antimicrobial compounds, such as bacteriocins, H_2_O_2_, or D- and L-lactate [4, 19]. On the other hand, in vivo, bacteria within the same microenvironment may influence each other’s metabolism and antimicrobial activity. Production of antimicrobial compounds in the presence of competing bacteria offers an evolutionary advantage but may incur a cost in the absence of competition [20]. For example, the basal bacteriocin production of the vaginal isolate *L. gasseri* EV1461 was 0-160 bacteriocin unit/ml (BU/ml), while after coincubation with three different *Lactobacillus* spp. or *Propionibacterium avium*, the bacteriocin production reached 1280–2560 BU/ml [21]. Similarly, bacteriocin production in *L. acidophilus* La-5 was stimulated by coculturing with viable, but not autoclaved, *Streptococcus thermophilus* and *L. delbrueckii* subsp. *bulgaricus* [22]. *L. plantarum* J23 also showed inducible bacteriocin production against viable *Oenococcus oeni*, as well as various *Lactobacillus* and *Pediococcus* strains [23]. This context-dependent, quorum sensing-mediated production of antimicrobials is well-documented among lactobacilli [24–26], and can be one of the explanations of the observed inhibitory differences detected by the two methods.

Altogether, our study suggests that cocultures that may reflect in vivo microbial interactions better should also be used to evaluate *Lactobacillus*-mediated inhibition of *G. vaginalis* growth. Our direct qPCR method enables rapid and quantitative measurement of antimicrobial activity in cocultures.

### Limitations

Our qPCR method was tested using NYC-III medium. While the direct qPCR performed well with this medium, employing a more diverse range of media would better demonstrate the robustness of the method. Another limitation is the number of lactobacilli tested. Analyzing additional strains would clarify whether the difference in inhibitory activity mediated by cell-free supernatant versus coculture is common among lactobacilli.

## Data Availability

Data is available upon reasonable request from the corresponding author.
